# The Relationship between the Serum NLRP1 Level and Coronary Lesions in Patients with Coronary Artery Disease

**DOI:** 10.1155/2023/2250055

**Published:** 2023-05-11

**Authors:** Jing Zong, Yixiao Wang, Siyu Pan, Yiming Yang, Jingfeng Peng, Fangfang Li, Luhong Xu, Shanshan Li, Wenhao Qian

**Affiliations:** ^1^Department of Cardiology, The Affiliated Hospital of Xuzhou Medical University, Xuzhou 221000, Jiangsu, China; ^2^Institute of Cardiovascular Disease, Xuzhou Medical University, Xuzhou 221000, Jiangsu, China

## Abstract

**Background:**

The pathogenesis of coronary artery disease is complex, and inflammation is one of the regulatory factors. The nucleotide-binding oligomerization domain (NOD)-like receptor protein 1 (NLRP1) plays an important role in the cellular inflammatory response, cell apoptosis, cell death, and autoimmune diseases. Whether the level of NLRP1 is related to the severity of coronary artery stenosis in patients with coronary artery disease (CAD) has not been reported.

**Objective:**

To test the serum level of NLRP1 in unstable angina (UA) patients and investigate the effect of NLRP1 on coronary stenosis severity of the coronary artery disease (CAD).

**Methods:**

307 patients hospitalized in the Department of Cardiology of the Affiliated Hospital of Xuzhou Medical University for coronary angiography from January 1, 2021, to December 31, 2022 were included. We detect the level of NLRP1 in the serum of the included patients. Patients were divided into UA group and control group according to coronary angiography results and other clinical data. We use logistic regression to screen the influencing factors of UA. Then, subgroups were divided according to the Gensini score and the number of coronary artery lesions, and the difference of serum NLRP1 level between the groups was compared. Spearman correlation analysis was used to explore the correlation between the serum NLRP1 level and Gensini score. We analyze the diagnostic value of NLRP1 for UA by drawing ROC curve.

**Results:**

The median level of serum NLRP1 in patients with UA (*n* = 257) was 49.71 pg/ml, IQR 30.15, 80.21, and that in patients without UA (*n* = 50) was 24.75 pg/ml, IQR 13.49, 41.95. Serum NLRP1 levels were significantly different among different subgroups. The patient's Gensini score was correlated with the patient's serum NLRP1 level.

**Conclusion:**

The serum NLRP1 level is increased in patients with UA, which is increased with the increasing severity of coronary lesions.

## 1. Introduction

Cardiovascular disease is one of the main causes of global incidence rate and mortality. According to the statistics of the World Health Organization, atherosclerotic cardiovascular disease (ASCVD) has become one of the main diseases threatening human health, with high morbidity, rapid progress, and high mortality as the main characteristics [1]. Inflammation is involved in the occurrence and development of atherosclerosis [2, 3]. With the in-depth and comprehensive study of apoptotic bodies and autophagosomes, inflammatory bodies are increasingly favored by researchers. The nucleotide-binding oligomerization domain (NOD)-like receptor protein 1 (NLRP1, also known as NALP1, DEFCAP, NAC, and CARD7) can activate caspase-1 and promote the inflammatory cytokine interleukin-1*β* (IL-1*β*) and interleukin-18 (IL-18) mature and aggravate inflammatory reaction [4]. The NLRP1 inflammatory body is the first inflammatory body found in human THP-1 monocytes. It is composed of NLRP1, caspase-1, caspase-5, junction protein ASC, and CARDINAL. It forms the first line of defense of the host by activating inflammatory cytokines [5]. There is evidence that coronary atherosclerotic heart disease is the result of multiple signal transduction pathways [6]. It was found that the knockout of NLRP1 gene alleviates the occurrence and development of myocardial hypertrophy and inflammation in mice [7, 8]. However, whether NLRP1 will play a similar role in the occurrence and development of coronary atherosclerosis and whether the level of NLRP1 is positively related to the degree of coronary stenosis in patients with coronary artery disease have not been reported yet. In this study, we determined the influence of the NLRP1 level on the degree of coronary stenosis and prognosis of patients with coronary artery disease by detecting the level of NLRP1 in the serum of patients with coronary artery disease, in order to explore new targets and new strategies for the prevention and treatment of coronary atherosclerotic artery disease.

## 2. Patients and Methods

### 2.1. Study Population

**Figure 1:**
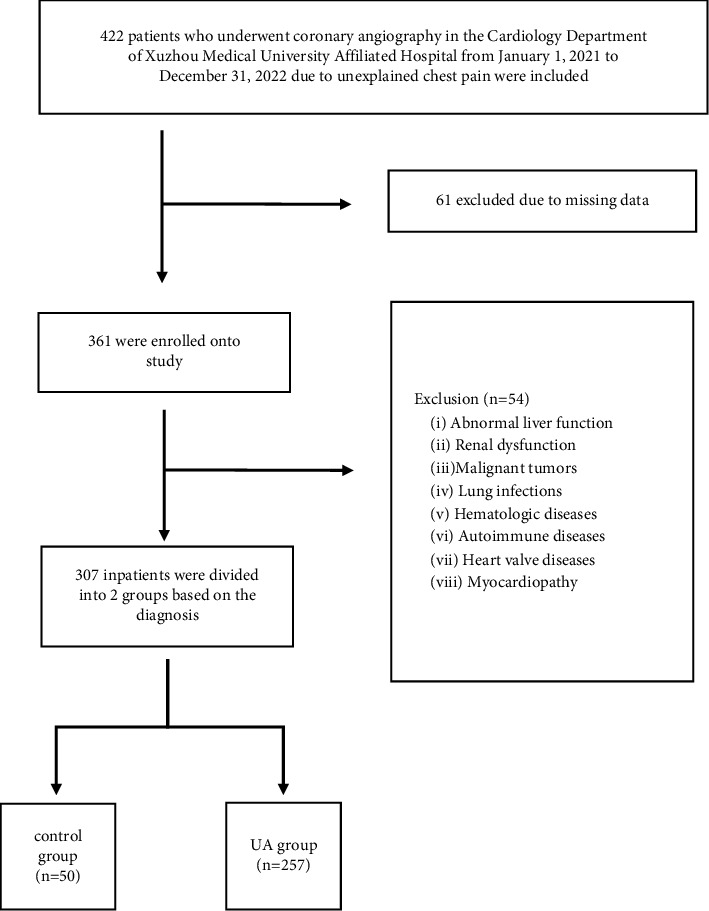
Flowchart of study design and recruited population. UA = unstable angina.

This study continuously included 307 patients who underwent coronary angiography in the Cardiology Department of Xuzhou Medical University Affiliated Hospital from January 1, 2021, to December 31, 2022, due to unexplained chest pain (Figure 1). All patients should complete relevant examinations such as blood routine, liver and kidney functions, coagulation functions, electrocardiogram, chest CT, and cardiac ultrasound before surgery. The preoperative evaluation of the patient was based on the above auxiliary examinations, and surgical contraindications such as severe liver and kidney dysfunction, electrolyte disorders, coagulation dysfunction, and iodine contrast agent allergy were ruled out. The patients with lung infection, malignant tumor, hematologic diseases, liver and kidney insufficiency, autoimmune disease, heart valve disease, myocardiopathy, data loss, and failure to do relevant examinations were not included in this study. According to the results of coronary angiography and clinical data, CAD is diagnosed when there is at least one major epicardial vessels stenosis diameter ≥50%. The 257 patients were diagnosed with UA through clinical manifestation, angiography, and other clinical examinations [9]. The control group consisted of 50 patients with unexplained chest pain. They received coronary angiography, but the results did not meet the diagnostic criteria for CAD. All patients agreed to undergo angiography and plasma collection. Blood samples were collected during cardiac catheterization before angiography. According to the Gensini scoring system specified by the American Heart Association and the severity of stenosis and corresponding coefficients of each coronary artery were calculated [10]. The subjects were divided into three groups according to Gensini score, namely, low-risk group (Gensini score < 30 points, *n* = 148), medium-risk group (30 ≤ Gensini score < 60 points, *n* = 66), and high-risk group (Gensini score ≥ 60 points, *n* = 43). According to the results of coronary angiography, the number of branches of the three main arteries [(eft anterior descending artery (LAD), left circumflex artery (LCX), and right coronary artery (RCA)) involved in the lesion were divided into control group (*n* = 50), single vessel lesion group (*n* = 88), and multivessel lesion group (*n* = 169). Since the left main coronary artery (LMCA) is the main branch of LAD and LCX, LMCA lesions are considered as two branch lesions. The severity of coronary artery disease was evaluated by Gensini score and the number of coronary artery lesions. The study was conducted in accordance with the Helsinki Declaration and approved by the Ethics Committee of the Affiliated Hospital of Xuzhou Medical University (No: XYFYLW2017-002) [11].

### 2.2. Methods

Patients' general data (gender, age, BMI, smoking history, alcohol history, hypertension history, and diabetes history), laboratory data (platelets, neutrophils, lymphocytes, monocytes, C-reactive protein, aspartate aminotransferase, alanine aminotransferase, total bilirubin, fibrinogen, glutamine transpeptidase, urea, creatinine, uric acid, cholesterol, triglyceride, high-density lipoprotein, low-density lipoprotein, and lipoprotein-a), statin use, aspirin use, and Imaging data, etc., were analyzed to compare the difference of clinical data among different groups of patients. All the subjects collected venous blood on an empty stomach within 24 hours after admission and tested it in the Biochemical Department of the Affiliated Hospital of Xuzhou Medical University. The Enzyme-linked Immunosorbent Assay (ELISA) was used to detect the serum NLRP1 level in enrolled patients. Patients who were tested for serum NLRP1 levels experienced discomfort symptoms such as chest pain within 24 hours before blood sampling. The ELISA kit was purchased from CUSABIO BIOTECH CO., Ltd, and the serum NLRP1 level was detected in strict accordance with the user manual [12]. To analyze the difference of serum NLRP1 level between UA patients and non-UA patients, the relationship between serum NLRP1 level and Gensini score, the relationship between serum NLRP1 level and the number of coronary artery lesions, and the correlation between serum NLRP1 level and Gensini score were considered.

### 2.3. Statistical Methods

First, Shapiro–Wilk test was used to test the normality of the measurement data. If it obeys the normal distribution, it is represented by *X* ± SD. If the normal distribution is not obeyed, the median (interquartile interval, IQR) shall be used. Classification variables are expressed in frequency (percentage). The variables between control group and UA group, each Gensini scoring group, and each coronary artery disease branch group were compared with ANOVA, nonparametric test, and chi-square test, correcting the results of intergroup comparisons using the FDR method. Logistic regression analysis is used to screen for risk factors of UA onset. Spearman correlation analysis was used to explore the correlation between the serum NLRP1 level and Gensini score. We draw the ROC curve to explore the predictive value of NLRP1 for UA.

## 3. Results

### 3.1. Comparison of Control Group and UA Patients

After screening, a total of 307 people were included in this study, with an average age of 64.08 ± 10.26 years, of whom 57.98% were male (median Gensini score was 20.0, IQR 8.0, 41.0). Among them, 257 people were patients with UA (median Gensini score was 24.0, IQR 14.3, 46.0) and 50 people were patients without UA (median Gensini score was 5.0, IQR 2.9, 7.0). The age (66.0 years old IQR 57.0, 72.0), the prevalence of diabetes (30.7%), serum fibrinogen (2.93 g/L, IQR 2.48, 3.27), the Gensini score (24.0, IQR 14.3, 46.0), and serum NLRP1 (49.71 pg/ml, IQR 30.15, 80.21) levels of UA patients were significantly higher than those of non-UA patients (the age was 62.5 years old, IQR 53.0, 68.25; the prevalence rate of diabetes was 12%; the median serum fibrinogen was 2.67 g/L, IQR 2.29, 2.95; the median Gensini score was 5.0, IQR 2.9, 7.0; the median serum NLRP1 was 24.75 pg/ml, IQR 13.49, 41.95), with statistically significant differences (*P* < 0.05). Detailed baseline characteristics for the study population are reported in Table 1.

### 3.2. Multivariate Logistic Analysis of Unstable Angina

NLRP1, age, diabetes, TG, LDL-C, FIB, and NE were used for multivariate binary logistic analysis. Multivariate logistic regression analysis was conducted based on whether UA occurred: yes = 1, no = 0. The results showed that NLRP1 was an independent risk factor for UA (*P* < 0.05), and its level was positively correlated with the occurrence of UA, as shown in Table 2.

### 3.3. Serum NLRP1 Level and the Severity of Coronary Atherosclerosis

According to the results of coronary angiography, a total of 257 patients (median Gensini score 24.0, IQR 14.3, 46.0) can be diagnosed as UA. The mean number of coronary artery lesions was 1.66 ± 0.48. In all grouping methods, the serum NLRP1 level (median 24.75 pg/ml, IQR 13.49, 41.95) of patients with non-UA was always the lowest and had statistically significant difference with other groups (*P* < 0.05).

#### 3.3.1. Serum NLRP1 Level and Gensini Score

**Figure 2:**
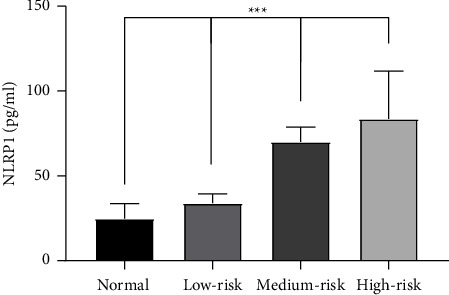
The serum NLRP1 level in each Gensini score grouping.

According to the analysis after grouping according to Gensini score, the serum NLRP1 level in the control group (median 24.75 pg/ml, IQR 13.49, 41.95) is the lowest, while the serum NLRP1 level in the high-risk group (median 83.57 pg/ml, IQR 63.39, 131.18) is the highest. The median level of serum NLRP1 in low-risk group and medium-risk group was 34.64 pg/ml, IQR 24.12, 56.14, and 69.99 pg/ml, IQR 53.13, 87.68, respectively. With the increase of Gensini score, the serum NLRP1 level of patients increased, and the difference of serum NLRP1 level between groups was statistically significant (*P* < 0.05). Using the FDR method to correct the results of intergroup comparisons, the results still have statistical significance (Table 3, Figure 2).

#### 3.3.2. Serum NLRP1 Expression Level and Branch Number of Coronary Lesions

**Figure 3:**
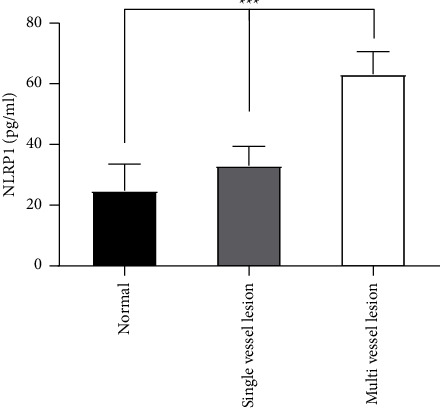
The serum NLRP1 level in each coronary lesion subgroup.

In the study of grouping based on the number of coronary artery lesions (Table 4, Figure 3). The serum NLRP1 level in the control group (median 24.75 pg/ml, IQR 13.49, 41.95) was still the lowest, while the serum NLRP1 level in the multivessel disease group (median 63.17 pg/ml, IQR 35.25, 91.20) was the highest. The median level of serum NLRP1 in patients with single vessel disease was 33.00 pg/ml, and IQR was 20.36, 57.72. With the increase of the number of coronary artery lesions, the serum NLRP1 level of patients increased, and the difference of serum NLRP1 level between groups was statistically significant. Using the FDR method to correct the results of intergroup comparisons, the results still have statistical significance.

### 3.4. Correlation Analysis of Serum NLRP1 Level and Gensini Score

**Figure 4:**
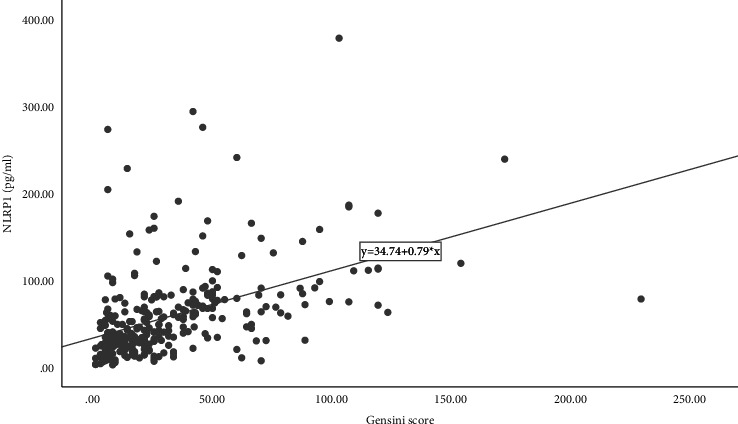
Correlation of serum nlrp1 levels and Gensini score.

Spearman correlation analysis of the serum NLRP1 level and Gensini score in patients with coronary artery disease showed that there was a correlation between serum the NLRP1 level and Gensini score (*ρ* = 0.580, *P* < 0.05).  Figure 4 shows a scatter plot based on NLRP1 and Gensini score.

(1) $$\begin{array}{c} y=34.74+0.79*x\text{,}\\ R^{2}\left(L\right)=0.224\text{.} \end{array}$$

### 3.5. Diagnostic Value of NLRP1 in UA

**Figure 5:**
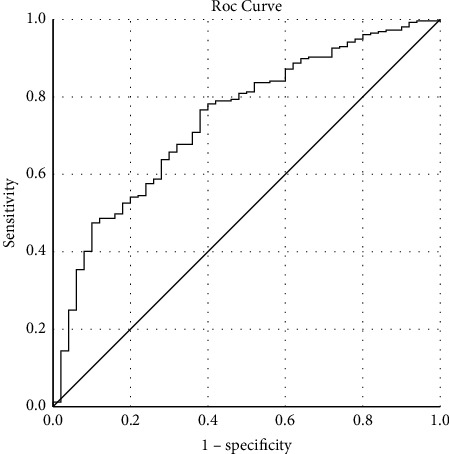
ROC curve of NLRP1 in diagnosis of UA.

The AUC of NLRP1 in diagnosing UA was 0.741 (95% CI (0.668–0.814)) (*P* < 0.001). When the best cut-off value of NLRP1 is 29.075 pg/L, the sensitivity and specificity of NLRP1 in diagnosing UA are 0.767 and 0.620, respectively (Figure 5).

## 4. Discussion

This experiment was based on the hospital to explore the correlation between the serum NLRP1 level and the severity of coronary atherosclerosis in patients undergoing coronary angiography. To investigate the relationship between the severity of coronary atherosclerosis and serum NLRP1 levels in patients with coronary artery disease, we selected unstable angina, which is a common type of coronary artery disease. According to our results, the serum NLRP1 level in patients with UA was significantly higher than that in patients without UA. Through binary logistic regression analysis, we found that NLRP1 is an independent risk factor for the occurrence of unstable angina. According to the patients' coronary arteriosclerosis Gensini score, there were differences in serum NLRP1 levels among the control group, low-risk group, medium-risk group, and high-risk group. The serum NLRP1 level of patients showed an obvious upward trend with the increase of Gensini score. Similarly, according to the number of coronary diseased branches, the serum NLRP1 level increased with the increase of the number of coronary diseased branches. The serum NLRP1 level in patients with multivessel disease was significantly higher than that in control group and patients with single vessel disease. The level of serum NLRP1 is positively correlated with the severity of coronary atherosclerosis. Because the serum NLRP1 level may change due to the patients suffering from certain inflammatory diseases, autoimmune diseases, blood diseases, malignant tumors, liver and kidney dysfunction, and other diseases, this experiment has not been included in this population. However, the experimental results are still significant to a considerable extent, which further indicates that the experimental results are reliable.

Coronary atherosclerotic heart disease is a relatively common type of cardiovascular disease, which refers to the heart disease that coronary atherosclerotic lesions or vasospasm cause coronary artery lumen stenosis or occlusion, and then cause myocardial ischemia, hypoxia, or necrosis, as referred to as coronary artery disease. Age, smoking, body mass index, dyslipidemia, hypertension, diabetes, and hyperhomocysteinemia (HCY) are important risk factors affecting the occurrence and development of coronary artery disease. The pathogenesis of coronary artery disease is complex, which is the result of many factors. Among them, inflammation is one of the regulatory factors of myocardial infarction pathology [13]. After myocardial infarction and necrosis death of myocardial cells, cardiac repair/remodeling can cause inflammatory reaction, leading to some important structural changes in the infarcted area and distal end. The degree and duration of inflammation can change the subsequent development process. In addition to activating inflammation and destroying myocardial tissue, migrated neutrophils and classically activated macrophages also alternately activate macrophages and myocardial myosin for fibrosis repair and reducing inflammatory response [14, 15].

Inflammatory corpuscles belong to the pattern recognition receptors (PRRs) family, called NOD-like receptors (NLRs). They are multiprotein complexes and members of the cytoplasmic protein family. They promote the production of caspase-1 dependent bioactive IL-1*β* through the formation of inflammatory corpuscles, thereby promoting the secretion of IL-1*β* and the downstream inflammatory response [4]. It has been confirmed that NLRP1 is associated with a variety of autoimmune diseases, such as enteritis, Addison's disease, vitiligo, type I diabetes, and rheumatoid arthritis [16, 17]. NLRP1 also plays an important role in nervous system diseases and is involved in the pathogenesis of acute angle closure glaucoma [18, 19]. NLRP1 is also an innate immune sensor, which produces IL-18 under metabolic stress and inhibits obesity and metabolic syndrome [20]. Studies have confirmed that apoptosis plays an important role in the occurrence and development of myocardial infarction [21, 22]. NLRP1 is the first member of the CED-4 family of apoptotic proteins found, which is essential for the initiation of programmed cell death in developing nematodes [23]. Related studies have confirmed that overexpression of NLRP1 in mammalian cells can lead to apoptosis [24]. NLRP1 plays an important role in cellular inflammation, apoptosis, cell death, and autoimmune diseases. NLRP1 is closely related to apoptosis and inflammation signal pathway. Some studies have shown that inhibiting NLRP1 can reduce the inflammatory pathway and disease progression of some nervous system diseases [25–27]. In stroke patients, the expression of NLRP1 will increase and will activate the inflammatory reaction and eventually lead to neuronal death [28]. As for the pathogenesis of coronary atherosclerosis, the current mainstream is the endothelial injury response theory, which involves a variety of inflammatory cells, secretes a variety of growth factors and proinflammatory mediators, and ultimately leads to coronary stenosis. Once there is a contradiction between coronary blood supply and myocardial blood demand, coronary blood flow cannot meet the needs of myocardial metabolism, which can cause myocardial ischemia and hypoxia. Temporary ischemia and hypoxia cause angina pectoris, while continuous severe myocardial ischemia can cause myocardial necrosis, namely, myocardial infarction. The process of coronary atherosclerosis and coronary artery disease is closely related to inflammation, but the relationship between NLRP1 and coronary atherosclerosis needs further exploration. Whether NLRP1 can directly or indirectly protect or damage vascular endothelial cells at the physiological and pathological levels is still unknown. At present, there are few studies on NLRP1 and coronary arteriosclerosis, and there is no systematic and comprehensive elaboration on its mechanism. The complex relationship network between NLRP1 and coronary atherosclerosis needs to be further explored, and the correlation between serum NLRP1 and coronary atherosclerosis needs to be further clarified. In previous research, our research team found that the knockout of NLRP1 gene will inhibit MAPKs and NF- *κ* B signal pathway, thereby reducing the occurrence and development of myocardial hypertrophy and inflammation in mice [7, 8]. Compared to NLRP1, research on NLRP3 is relatively extensive. NLRP3 has been considered to be related to autoimmune diseases, tumors and, of course, coronary atherosclerosis. The triggering and activation mechanisms of NLRP3 activation are usually consistent among different species. However, the situation of NLRP1 in humans and mice may not be the same, as they exhibit significant differences, which makes NLRP1 more important in clinical research [29, 30]. But up to now, there is no clinical study involving the relationship between coronary atherosclerosis and NLRP1.

The advantage of this study is that the number of coronary artery lesions and Gensini score is used to represent the severity of coronary atherosclerosis. The Gensini score combined with the number of coronary artery lesions to evaluate and express the severity of coronary atherosclerosis is more reliable and objective than a single indicator. The results suggest that NLRP1 is closely related to the severity of coronary artery disease in patients with UA. It can lay a clinical foundation for the later study of the role and molecular mechanism of NLRP1 on cardiac remodeling and heart failure in patients with CAD and has strong feasibility in theory. The test method is easy to learn, not complicated, and can be popularized. Our study laid the foundation for exploring the relationship between NLRP1 and coronary atherosclerosis and provided objective evidence support. It has important clinical significance to provide a new perspective and direction for the pathogenesis and treatment of atherosclerosis and its related diseases. The results can provide ideas for exploring new targets and strategies for the prevention and treatment of coronary atherosclerotic heart disease.

Based on the baseline characteristics of this study, there is no difference in multiple cardiovascular risk factors between patients with non-UA and patients with UA [31], which may be due to the failure to control the drug use of patients in the experimental design. Most patients have begun to receive drug treatment before hospitalization or coronary angiography, which affects some of the patients' laboratory indicators. However, whether the use of drugs has an impact on the serum NLRP1 level of patients needs further research to confirm. Therefore, this is the limitation of this experiment. Secondly, our assessment of the severity of coronary atherosclerosis is based on coronary angiography, which is unethical for normal people. Therefore, we included patients with chest pain and suspected coronary artery disease. This may significantly reduce the sample size of the control group.

## 5. Conclusion

The level of serum NLRP1 in patients with UA was significantly higher than that in patients without UA. NLRP1 is closely related to coronary atherosclerosis and it can serve as an effective indicator for predicting UA.

## Figures and Tables

**Table 1:** Comparison of basic data between patients with UA and non-UA.

Variable	Control (*n* = 50)	UA (*n* = 257)	*P* value
Age (IQR)	62.5 (53.0, 68.25)	66.0 (57.0, 72.0)	0.049
Sex (male, *n*, %)	22 (44.0)	148 (55.6)	0.077
BMI (kg/m^2^, median, IQR)	24.9 (22.5, 27.8)	25.3 (22.9, 27.2)	0.804
Tobacco use (yes, *n*, %)	16 (32.0)	69 (26.8)	0.456
Alcohol consumption (yes, *n*, %)	11 (22.0)	56 (21.8)	0.974
Hypertension (yes, *n*, %)	29 (58.0)	162 (63.0)	0.502
Diabetes (yes, *n*, %)	6 (12.0)	79 (30.7)	0.007
TC (mmol/L, IQR)	4.33 (3.39, 5.07)	4.24 (3.59, 4.86)	0.676
TG (mmol/L, IQR)	1.37 (0.94, 1.80)	1.54 (1.07, 1.92)	0.196
HDL-C (mmol/L, IQR)	1.10 (0.91, 1.32)	1.05 (0.91, 1.18)	0.201
LDL-C (mmol/L, IQR)	2.58 (1.77, 3.22)	2.45 (1.88, 2.88)	0.453
LP (a) (mg/L, IQR)	209 (134.5, 345.0)	298.0 (177.5, 344.5)	0.115
AST (U/L, IQR)	19.0 (16.0, 24.0)	18.0 (15.0, 24.0)	0.661
ALT (U/L, IQR)	18.0 (13.0, 25.5)	16.0 (12.0, 26.0)	0.425
TBIL (*μ*mol/L, IQR)	11.75 (8.78, 16.85)	12.2 (9.3, 13.8)	0.729
FIB (g/L, IQR)	2.67 (2.29, 2.95)	2.93 (2.48, 3.27)	0.017
PLT (10^9^/L, IQR)	210.5 (169.25, 242.0)	213.0 (178.0, 246.0)	0.715
NE (10^9^/L, IQR)	3.41 (2.89, 4.23)	3.79 (2.91, 4.62)	0.192
LY (10^9^/L, IQR)	1.6 (1.2, 2.1)	1.6 (1.3, 1.9)	0.906
MO (10^9^/L, IQR)	0.39 (0.33, 0.49)	0.39 (0.30, 0.50)	0.901
CRP (mg/L, IQR)	1.10 (0.50, 3.55)	1.50 (0.50, 4.15)	0.417
GGT (U/L, IQR)	21.0 (16.0, 29.3)	22.0 (15.0, 30.0)	0.890
Urea (mmol/L, IQR)	5.42 (4.68, 6.28)	5.27 (4.40, 6.46)	0.810
CREA (*μ*mol/L, IQR)	62.5 (54.0, 68.25)	63.0 (55.0, 71.0)	0.510
UA (*μ*mol/L, IQR)	297.0 (242.8, 324.3)	300.0 (242.5, 347.5)	0.888
Statin (yes, *n*, %)	16 (32.0)	96 (37.4)	0.472
Aspirin (yes, *n*, %)	19 (38.0)	120 (46.7)	0.259
NLRP1 (pg/ml, IQR)	24.75 (13.49, 41.95)	49.71 (30.15, 80.21)	≤0.001
Gensini score (IQR)	5.0 (2.9, 7.0)	24.0 (14.3, 46.0)	≤0.001

BMI: body mass index, TC: total cholesterol, TG: triglyceride, HDL-C: high-density lipoprotein-cholesterol, LDL-C: low-density lipoprotein-cholesterol, LP (a): lipoprotein-a, AST: aspartate aminotransferase, ALT: alanine aminotransferase,TBIL: total bilirubin, FIB: fibrinogen, PLT: platelet, NE: neutrophilicgranulocyte, LY: lymphocyte, MO: monocytes, CRP: C-reactive protein GGT: gamma glutamyl transferase, CREA: creatine, and UA: uric acid.

**Table 2:** Logistic regression analysis of unstable angina.

Variable	*β*	SE	Wald	OR (95% CI)	*P* value
NLRP1	0.021	0.006	10.547	1.021 (1.008–1.034)	0.001
Age	0.028	0.017	2.857	1.029 (0.996–1.063)	0.091
Diabetes	0.831	0.478	3.027	2.295 (0.900–5.853)	0.082
TG	0.251	0.223	1.268	1.286 (0.830–1.991)	0.260
LDL-C	−0.191	0.200	0.908	0.826 (0.558–1.223)	0.341
FIB	0.388	0.288	1.814	1.475 (0.838–2.595)	0.178
NE	0.084	0.134	0.390	1.087 (0.836–1.415)	0.533

TG: triglyceride, LDL-C: low-density lipoprotein-cholesterol, FIB: fibrinogen, and NE: neutrophilicgranulocyte.

**Table 3:** Comparison of serum NLRP1 levels based on Gensini score grouping.

Variable	Control (*n* = 50)	Low-risk (*n* = 148)	Medium-risk (*n* = 66)	High-risk (*n* = 43)	*P* value
NLRP1 (pg/L, IQR)	24.75 (13.49, 41.95)	34.64 (24.12, 56.14)	69.99 (53.13, 87.68)	83.57 (13.49, 41.95)	≤0.001

FDRp1 = 0.002 (comparison of serum NLRP1 levels between control group and low-risk group). FDRp2 ≤ 0.001 (comparison of serum NLRP1 levels between control group and medium-risk group). FDRp3 ≤ 0.001 (comparison of serum NLRP1 levels between control group and high-risk group). FDRp4 ≤ 0.001 (comparison of serum NLRP1 levels between low-risk group and medium-risk group). FDRp5 ≤ 0.001 (Comparison of serum NLRP1 levels between Low-risk group and High-risk group). FDRp6 = 0.024 (comparison of serum NLRP1 levels between medium-risk group and multihigh-risk group).

**Table 4:** Comparison of serum NLRP1 levels based on coronary lesion branch number grouping.

Variable	Control (*n* = 50)	Single vessel lesion (*n* = 88)	Multi vessel lesion (*n* = 169)	*P* value
NLRP1 (pg/L, IQR)	24.75 (13.49, 41.95)	33.00 (20.36, 57.72)	63.17 (35.25, 91.20)	≤0.001

FDRp1 = 0.014 (comparison of serum NLRP1 levels between control group and single vessel lesion group). FDRp2 ≤ 0.001 (comparison of serum NLRP1 levels between control group and multivessel lesion group). FDRp3 ≤ 0.001 (comparison of serum NLRP1 levels between single vessel lesion group and multivessel lesion group).

## Data Availability

The datasets used to support the findings of this study available from the corresponding author upon reasonable request.
